# Oxymatrine induces human pancreatic cancer PANC-1 cells apoptosis via regulating expression of Bcl-2 and IAP families, and releasing of cytochrome c

**DOI:** 10.1186/1756-9966-30-66

**Published:** 2011-06-29

**Authors:** Qi Ling, Xiao Xu, Xuyong Wei, Weibing Wang, Bin Zhou, Bei Wang, Shusen Zheng

**Affiliations:** 1 Division of Hepatobiliary and Pancreatic Surgery, Department of Surgery, First Affiliated Hospital, Zhejiang University School of Medicine, 79 Qingchun Road, Hangzhou 310003, China

## Abstract

**Background:**

Oxymatrine, an isolated extract from traditional Chinese herb *Sophora Flavescens Ait*, has been traditionally used for therapy of anti-hepatitis B virus, anti-inflammation and anti-anaphylaxis. The present study was to investigate the anti-cancer effect of oxymatrine on human pancreatic cancer PANC-1 cells, and its possible molecular mechanism.

**Methods:**

The effect of oxymatrine on the viability and apoptosis was examined by methyl thiazolyl tetrazolium and flow cytometry analysis. The expression of Bax, Bcl-2, Bcl-x (L/S), Bid, Bad, HIAP-1, HIAP-2, XIAP, NAIP, Livin and Survivin genes was accessed by RT-PCR. The levels of cytochrome c and caspase 3 protein were assessed by Western blotting.

**Results:**

Oxymatrine inhibited cell viability and induced apoptosis of PANC-1 cells in a time- and dose-dependent manner. This was accompanied by down-regulated expression of Livin and Survivin genes while the Bax/Bcl-2 ratio was upregulated. Furthermore, oxymatrine treatment led to the release of cytochrome c and activation of caspase-3 proteins.

**Conclusion:**

Oxymatrine can induce apoptotic cell death of human pancreatic cancer, which might be attributed to the regulation of Bcl-2 and IAP families, release of mitochondrial cytochrome c and activation of caspase-3.

## Background

Pancreatic cancer is one of the most common malignant tumors worldwide. It can only be managed with surgical treatment in limited cases, whereas the majority of cases presented advanced tumors responds poorly to current available medical therapies [1]. Similar to other tumor types, insufficient cell death and/or excessive proliferation appears to be a major unfavorable feature of pancreatic cancer [2]. Investigations in inducing programmed cell death and deepening the understanding of molecular mechanisms may provide important value to develop new therapeutic options.

*Sophora flavescens ait *(kushen), a traditional Chinese herb, has been used as folk medicine for many kinds of diseases. As one of the major components of *Sophora flavescens ait*, oxymatrine has exhibited various pharmacological effects such as anti-hepatitis virus infection, anti-hepatic fibrosis, anti-inflammation, anti-anaphylaxis and other immune-regulation [3-6]. Some previous studies have also reported anti-cancer activity of oxymatrine in human gastric cancer cells and human breast cancer cells [7,8]. In the present study, we aim to determine the anti-cancer effect of oxymatrine on human pancreatic cancer cells and to further clarify its possible molecular mechanism.

## Methods

### Materials

RPMI 1640 medium was obtained from Gibco BRL. Newborn bovine serum was supplied by Sijiqing Biotechnology Co. (Hangzhou, China). Monoclonal antibodies to Bcl-2, Bax, Bid, Bad, Bcl-x (L/S), HIAP-1, HIAP-2, XIAP, NAIP, Livin, Survivin, cytochrome c, caspase 3 and β-actin were purchased from Cell Signal, USA. Oxymatrine was purchased from the National Institute for Pharmaceutical and Biological Products, Beijing, China. The drug was dissolved in DMSO with the stock concentration of 10 mg/mL. It was further diluted in culture medium with the final DMSO concentration < 1%. 3-(4, 5-dimethylthiazol-2-yl)-2, 5-diphenyltetrazolium bromide (MTT) and propidium iodide (PI) were purchased from Sigma Chemical Corporation, USA.

### Cell culture

Human pancreatic cancer cell lines (PANC-1, BxPC-3 and AsPC-1) were provided by Cancer Institute of Zhejiang University. PANC-1, BxPC-3 and AsPC-1 cells were maintained in RPMI 1640 medium (Gibco BRL) supplemented with 10% heat-inactivated fetal bovine serum (Si-Ji-Qing Biotechnology Co, Hangzhou, China), 100 U/mL penicillin and 100 μg/mL streptomycin at 37°C in a 5% CO_2 _atmosphere.

### Cell viability assay

PANC-1, BxPC-3 and AsPC-1 cells (1 × 10^4 ^in 100 μL) were seeded on 96-well plates in triplicate respectively. Following a 24-h culture at 37 °C, the medium was replaced with fresh medium containing vehicle control or various concentrations of oxymatrine in a final volume of 200 μL. Cells were incubated at 37 °C for 24 h. Then 50 μL of MTT (2 mg/mL in PBS) was added to each well, incubated for an additional 4 h, the plate was centrifuged at 1000 r/min for 10 min, then the medium was removed. The MTT formazan precipitate was dissolved in 100 μL DMSO, shaken mechanically for 10 min and then read immediately at 570 nm by a plate reader (Opsys MR, Denex Technology, USA).

### Flow cytometry

PANC-1 cells were treated with different concentration of oxymatrine (0, 0.5, 1 and 2 mg/mL) for 48 h at cell density of 2 × 10^5 ^cells/mL, and then stained with Annexin V-FITC and PI (Sigma, USA). Annexin V-FITC positive and PI negative cells were considered as apoptotic cells.

### RT-PCR assay

PANC-1 cells 1 × 10^5 ^were seeded on 24-well plate. After 24-h culture, cells were treated with 0.5, 1, 2 mg/mL oxymatrine and vehicle for 48 h. Total RNA was extracted using Trizol (Invitrogen, USA). cDNA synthesis was performed using a RNA PCR kit (TaKaRA Biomedicals, Osaka, Japan) with the supplied oligo dT primer (Table 1). Samples were separated on 20 g/L agarose gel and visualized with ethidium bromide staining under UV light.

The PCR primer and regimen were as following: 5'-GTGGAGGAGCTCTTCAGGGA-3', 5'-AGGCACCCAGGGTGATGCAA-3' for Bcl-2 (304 bp, 42 cycles); 5'- GGCCCACCAGCTCTGAGCAGA-3', 5'- GCCACGTGGGCGGTCCCAAAGT -3' for Bax (479 bp, 42 cycles); 5'-CAGTGATCTGCTCCACATTC-3' 5'-TCCAGCTAGGATGATAGGAC-3' for Bad (340 bp, 40 cycles); 5'-GACCCGGTGCCTCAGGA-3', 5'-ATGGTCACGGTCTGCCA-3' for Bid (586 bp, 40 cycles); 5'-TTGGACAATGGACTGGTTGA-3', 5'-GTAGAGTGGATGGTCAGTG-3' for Bcl-X (l/s) (780/591 bp, 42 cycles); 5'-GCCTGATGCTGGATAACTGG-3', 5'-GGCGACAGAAAAGTCAATGG-3' for HIAP-1 (349 bp, 38 cycles); 5'-GCCTGATGCTGGATAACTGG-3', 5'-GCTCTTGCCAATTCTGATGG-3' for HIAP-2 (361 bp, 38 cycles); 5'-GTGACTAGATGTCCACAAGG-3', 5'-CTTGAGGAGTGTCTGGTAAG-3' for XIAP (368 bp, 38 cycles); 5'-TTATACCAGCGCCAGTTTCC-3', 5'-TGGTGGAACTAAGGGAGAGG-3' for NAIP (299 bp, 38 cycles); 5'-CTCCTTCTATGACTGGC-3', 5'-ACACTCAGCACAGACC-3' for Livin (496 bp, 38 cycles); 5'-CAGATTTGAATCGCGGGACCC-3', 5'-CCAAGTCTGGCTCGTTCTCAG-3' for Survivin (206 bp, 38 cycles); 5'-GGAGTCCTGTGGCATCCACG-3' 5'-CTAGAAGCATTTGCGGTGGA-3' for β-actin (322 bp, 30 cycles). The PCR conditions were denaturation at 94°C for 1 min, annealing at 56°C for 1 min, and extension at 72 °C for 2 min.

### Western blotting

PANC-1 cells (5 × 106) treated with 0.5, 1 and 2 mg/mL oxymatrine and vehicle respectively for 48 h were lysed by 4 g/L trypsin containing 0.2 g/L EDTA, then collected after washed twice with phosphatebuffered saline (PBS, pH 7.4). Total protein extract from PANC-1 cells was prepared using cell lysis buffer [150 mmol/L NaCl, 0.5 mol/L Tris-HCl (pH 7.2), 0.25 mol/L EDTA (pH 8.0), 10 g/L Triton X-100, 50 mL/L glycerol, 12.5 g/L SDS]. The extract (30 μg) was electrophoresed on 12 g/L SDS-PAGE and electroblotted onto polyvinylidene difluoride membrane (PVDF, Millipore Corp., Bedford, MA) for 2 h in a buffer containing 25 mmol/L Tris-HCl (pH 8.3), 192 mmol/L glycine and 200 mL/L methanol. The blots were blocked with 50 g/L nonfat milk in TBST washing buffer for 2 h at room temperature and then incubated at 4 °C overnight with antibodies. All antibodies were diluted in TBST according to the manufacturer's instructions. After washed at room temperature with washing buffer, the blots were labeled with peroxidase-conjugated secondary antibodies.

### Statistical analysis

SPSS for Windows version 11.0 (SPSS Inc., Chicago, IL) was used to complete all the analyses. Statistical significance was determined by Student's *t*-test. A *P *value of < 0.05 was considered statistically significant.

## Results

### Oxymatrine inhibiting PANC-1, BxPc-3 and AsPC-1cells viability

**Figure 1:**
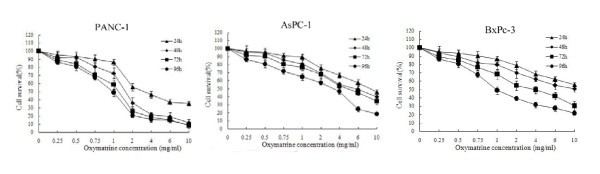
**The inhibitory effect of oxymatrine on the growth of PANC-1, BxPc-3 and AsPC-1cells**. The inhibitory effects of oxymatrine on the growth of PANC-1, BxPc-3 and AsPC-1 cells were observed in both a dose- and time-dependent manner. PANC-1, BxPc-3 and AsPC-1 cells treated with different concentrations of oxymatrine (0.25, 0.5, 1, 2, 4, 6 and 10 mg/mL) and the cell survival rates were calculated for different periods of time (24, 48, 72 and 96 h).

The inhibitory effect of oxymatrine on the growth of PANC-1, BxPc-3 and AsPC-1 cells was assessed by the MTT assay. The various concentrations of oxymatrine inhibited the viability of PANC-1, BxPc-3 and AsPC-1 cells in both a dose- and time-dependent manner (Figure 1). In these three cell lines, PANC-1 was the most sensitive cell line to oxymatrine. Thus in the following experiment, PANC-1 was used according to the MTT assay.

At the concentration of 0.5-2 mg/mL of oxymatrine, PANC-1 cells sharply decreased on viability. However, higher concentration of oxymatrine (> 2 mg/mL) had a saturated inhibitory effect. Thus we chose the concentration of 0.5, 1 and 2 mg/mL for further investigation of the molecular mechanism. During the following experiment at 48 h, oxymatrine showed a significantly higher inhibiting effect than that at 24 h. In contrast, there was no significant difference in cell survival among prolonged treatment for 72 h, and 96 h. Therefore, we choose the time point of 48 h for the further investigation.

### Oxymatrine inducing PANC-1 cells apoptosis

**Figure 2:**
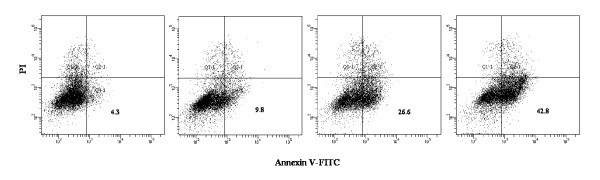
**Apoptosis analysis of PANC-1 cells**. Apoptosis analysis of PANC-1 cells induced by different concentration of oxymatrine (0, 0.5, 1 and 2 mg/ml; from left to right panel) for 48 h, using flow cytometer with Annexin V-FITC/PI binding assay.

Oxymatine-induced apoptotic cell death was found using Annexin V-FITC/PI double stained flow cytometry. Annexin V-FITC positive and PI negative cells, which were considered as early apoptotic cells, increased in a dose-dependent manner (Figure 2). Oxymatrine-treated PANC-1 had increased apoptosis rates at concentration of 1 and 2 mg/mL than the control group (*P *< 0.05).

### Oxymatrine regulating expression of Bcl-2 family

**Figure 3:**
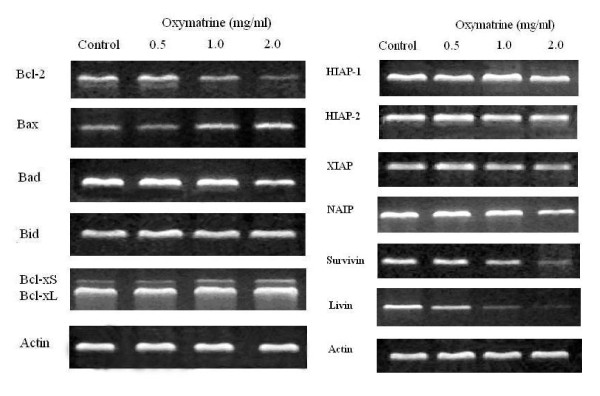
**The effect of oxymatrine on the mRNA expression of Bcl-2 and IAP family**. The effect of oxymatrine on the mRNA expression of Bcl-2 family and IAP family. PANC-1 cells were treated with different concentration (0, 0.5, 1 and 2 mg/ml) of oxymatrine for 48 h.

**Figure 4:**
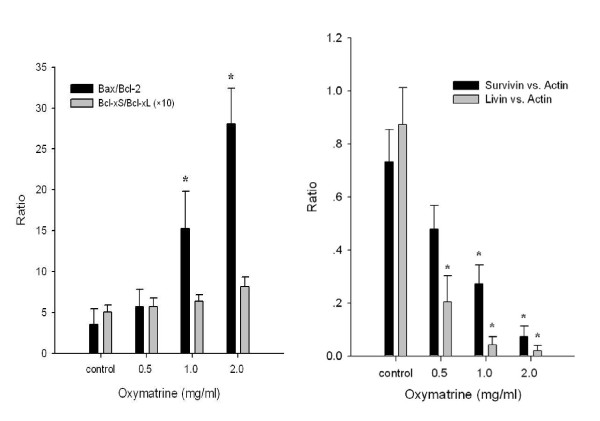
**The ratio of Bax/Bcl-2 changes and Survivin/Actin and Livin/Actin changes**. The ratio of Bax/Bcl-2 changes and Survivin/Actin and Livin/Actin changes after different treatments as determined by densitometric measurements, *: *P *< 0.05 as compared with controls.

The Bcl-2 mRNA expression was reduced when PANC-1 cells were exposed to 1.0 and 2.0 mg/mL oxymatrine compared with controls, while Bax and Bcl-xS mRNA expressions were increased (Figure 3A). A significant increase of Bax/Bcl-2 ratio was found in the oxymatrine treated (1.0 and 2.0 mg/mL) groups compared with controls as determined by densitometric measurements (*P *< 0.05) (Figure 4A). Although the Bcl-xS/Bcl-xL ratio increased in the oxymatrine treated groups compared with controls, no significant difference was noted (Figure 4A). Oxymatrine did not alter the expression of Bid and Bad mRNA levels (Figure 3A).

### Oxymatrine regulated expression of IAP family

Compared with controls, the Livin mRNA expression was remarkably down-regulated after treated with different concentrations of oxymatrine (all *P *< 0.05), while the level of Survivin mRNA expression did not decrease until PANC-1 cells were exposed to high concentrations (1.0 and 2.0 mg/mL) of oxymatrine (Figure 4B). In contrast, no apparent changes of HIAP-1, HIAP-2, XIAP and NAIP mRNA expressions were found at different levels of oxymatrine treated group compared with controls (Figure 3B).

### Oxymatrine releasing cytochrome c and activated caspase-3

**Figure 5:**
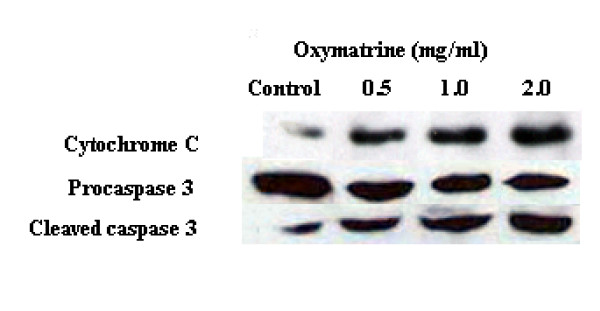
**The effect of oxymatrine on release of mitochondrial cytochrome c and activation of caspase-3**. The effect of oxymatrine on release of mitochondrial cytochrome c and activation of caspase-3. PANC-1 cells were treated with different concentration (0, 0.5, 1 and 2 mg/ml) of oxymatrine for 48 h. A 1% concentration of DMSO was used for control.

Oxymatrine treatment led to a dose-dependent release of cytochrome c and activation of caspase-3 (Figure 5). A remarkable increase of cytochrome c protein level was monitored after oxymatrine treatment. The cleaved caspase-3 protein was observed after treated with 0.5 mg/mL oxymatrine and then presented a sharp increase as treated with higher concentration of oxymatrine. Mitochondrial apoptotic pathway may be responsible for cell death characteristics induced by oxymatrine.

## Discussion

Insufficient or excessive cell death can lead to cancer [2]. Apoptosis plays an essential role for organ development, homeostasis, and immune defense and provides mechanisms for the anti-cancer therapies. In the present study, the growth and viability of human pancreatic cancer cells were largely inhibited by the extract of traditional Chinese herb oxymatrine. Furthermore, oxymatrine can induce cell apoptosis in human pancreatic cancer. As this pilot study would be extended to further cell lines and primary cultures, induction of apoptosis of pancreatic cancer with traditional Chinese anti-cancer drugs would be probably a promising approach of pancreatic cancer.

Multiple signal pathways are involved in the regulation of apoptosis and the molecular regulators have been identified. Among them, the Bcl-2 family plays a central role in the activation of caspases and dominates the regulation of apoptosis [9-11]. Some Bcl-2 family members can promote cell death, such as Bax, Bad, Bid, Bcl-xS while others promote cell survival, like Bcl-2, Bcl-xL. The relative balance between these anti- and pro-apoptotic Bcl-2 family members influences the susceptibility of cells to a death signal. In this study, oxymatrine-induced apoptotic cell death was involved in down-regulation of Bcl-2 and up-regulation of Bax. Bax directly or indirectly generates cell death signals while Bcl-2 is the dominant inhibitor of Bax. The Bax/Bcl-2 ratio has been reported to determine the eventual outcome (apoptosis or survival) [12]. Our result demonstrated about 5 and 9 fold Bax/Bcl-2 ratios at the treatment of 1.0 and 2 mg/ml concentration of oxymatrine respectively, compared with controls, which suggested that the alteration of Bax/Bcl-2 expression was associated with oxymatrine-induced pancreatic cancer cells apoptosis. Besides Bax/Bcl-2 ratio, the Bcl-xS/Bcl-xL ratio also plays a major role in the fate of the cell following an apoptotic stimulus. The dominant inhibitor Bcl-xS can abrogate Bcl-2 function via its binding to Bcl-2, which prevents Bcl-2 from interaction with Bax and thus leaves Bax unopposed in its cell-death effectors function [13]. Although Bcl-xS/Bcl-xL ratio appeared to be very important in deciding cell fate in a number of cell types [14-16], the role of Bcl-xL in pancreatic cell apoptosis is still unknown. In this study, Bcl-xS/Bcl-xL ratio was increased in oxymatrine treated groups compared with controls. However, no statistical significance was noted and whether the Bcl-xL gene is involved in the oxymatrine-induced apoptosis needs further verification.

Caspases are the central components in the apoptotic response. Both intrinsic (ie mitochondrial) and extrinsic (ie death receptor) pathways can activate caspases. In mitochondrion-dependent apoptosis, cytochrome c released from the mitochondria can activate the initiator caspase-9 and the effector caspase-3, which play key roles in both intrinsic and extrinsic pathways [17,18]. Bcl-2 exerts control of mitochondrial permeability and preventing the cytochrome C release while Bax can promote mitochondrial permeability. Thus the elevated Bax/Bcl-2 ratio would indicate the release of cytochrome c. The Western blotting analysis showed that a dose-dependent release of cytochrome c and activation of caspase-3 upon 48 h treatment was consistent with the PCR results. This study demonstrates that oxymatrine treatment leads to the release of cytochrome c and activation of caspase-3.

Apoptosis may also be inhibited by a variety of proteins including members of the inhibitors of apoptosis (IAP) family [19]. IAPs comprise a family of structurally similar proteins, such as HIAP-1, HIAP-2, XIAP, NAIP, Livin and Survivin, largely over-expressed by most tumors. They promote tumor cell survival after a wide variety of apoptotic stimuli elicited via intrinsic and extrinsic pathways [19]. Our results revealed that oxymatrine-induced apoptosis was related to down-regulation of Livin and Survivin expressions. Livin (BIRC7), a novel identified member of IAP family, selectively binds the endogenous IAP antagonist SMAC and caspase-3, caspase-7, and caspase-9, as a result, inhibits apoptosis [19-21]. Survivin can also bind the effector cell death proteases caspases-3 and -7 and inhibit caspase activity and cell death. Furthermore, Survivin-(hepatitis B X-interacting protein) complexes can bind pro-caspase-9 and selectively suppresses apoptosis via the mitochondria/cytochrome c pathway [19,22]. Livin and Survivin expressions were found in primary and cultured tumor cells and their overexpression was associated with poor prognosis [23-25]. In this study, Livin expression was markedly inhibited by oxymatrine in a dose-dependent manner, while the expression of Survivin was only down-regulated at a relative high dose of oxymatrine.

## Conclusions

In this study, a dose- and time-dependent oxymatrine-induced pancreatic cancer cell death via increasing pro-apoptotic Bax expression and decreasing anti-apoptotic Bcl-2 and Bcl-xS expression result in the release of cytochrome to cytosol, followed by activation of caspapse-3 and finally lead to cell apoptosis. Moreover, down-regulation of IAP family members (Livin and Survivin) is likely to be involved in the oxymatrine-induced apoptosis. These findings may provide a promising approach of pancreatic cancer's therapy based on traditional Chinese medicine.

## Competing interests

The authors declare that they have no competing interests.

## Authors' contributions

LQ proposed the study and wrote the first draft. WB analyzed the data. All authors contributed to the design and interpretation of the study and to further drafts. ZSS is the guarantor. All authors read and approved the final manuscript.
